# Multi-omics analysis the differences of VOCs terpenoid synthesis pathway in maintaining obligate mutualism between *Ficus hirta* Vahl and its pollinators

**DOI:** 10.3389/fpls.2022.1006291

**Published:** 2022-11-15

**Authors:** Songle Fan, Yongxia Jia, Rong Wang, Xiaoyong Chen, Wanzhen Liu, Hui Yu

**Affiliations:** ^1^ Key Laboratory of Plant Resource Conservation and Sustainable Utilization, South China Botanical Garden, Chinese Academy of Sciences, Guangzhou, China; ^2^ Guangdong Provincial Key Laboratory of Digital Botanical Garden, South China Botanical Garden, Chinese Academy of Sciences, Guangzhou, China; ^3^ University of Chinese Academy of Sciences, Beijing, China; ^4^ School of Ecological and Environmental Sciences, Tiantong National Station for Forest Ecosystem Research, East China Normal University, Shanghai, China

**Keywords:** *Ficus hirta* Vahl, proteome, terpenoid, transcriptome, VOCs

## Abstract

**Inroduction:**

Volatile organic compounds (VOCs) emitted by the receptive syconia of *Ficus* species is a key trait to attract their obligate pollinating fig wasps. *Ficus hirta* Vahl is a dioecious shrub, which is pollinated by a highly specialized symbiotic pollinator in southern China. Terpenoids are the main components of VOCs in *F. hirta* and play ecological roles in pollinator attraction, allelopathy, and plant defense. However, it remains unclear that what molecular mechanism difference in terpenoid synthesis pathways between pre-receptive stage (A-phase) and receptive stage (B-phase) of *F. hirta* syconia.

**Methods:**

Transcriptome, proteome and Gas Chromatography-Mass Spectrometer (GC-MS) were applied here to analyze these difference.

**Results and discussion:**

Compared to A-phase syconia, the genes (*ACAT2*, *HMGR3*, *GGPS2*, *HDR*, *GPS2*, *TPS2*, *TPS4*, *TPS10-4*, *TPS14*) related to the terpenoid synthesis pathway had higher expression level in receptive syconia (B-phase) according to transcriptome sequencing. Seven differentially expressed transcription factors were screened, namely *bHLH7*, *MYB1R1*, *PRE6*, *AIL1*, *RF2b*, *ANT*, *VRN1*. Specifically, *bHLH7* was only specifically expressed in B-phase. 235 differentially expressed proteins (DEPs) were mainly located in the cytoplasm and chloroplasts. Kyoto Encyclopedia of Genes and Genomes (KEGG) analysis showed that the DEPs were mainly enriched in the metabolic process. A total of 9 terpenoid synthesis proteins were identified in the proteome. Among them, 4 proteins in methylerythritol phosphate (MEP) pathway were all down-regulated. Results suggested the synthesis of terpenoids precursors in B-phase bracts were mainly accomplished through the mevalonic acid (MVA) pathway in cytoplasm. Correlation analysis between the transcriptome and proteome, we detected a total of 1082 transcripts/proteins, three of which are related to stress. From the VOCs analysis, the average percent of monoterpenoids emitted by A-phase and B-phase syconia were 8.29% and 37.08%, while those of sesquiterpenes were 88.43% and 55.02% respectively. Monoterpenes (camphene, myrcene, camphor, menthol) were only detected in VOCs of B-phase syconia. To attract pollinators, B-phase syconia of *F. hirta* need more monoterpenoids and less sesquiterpenes. We speculate that transcription factor *bHLH7* may regulate the terpenoid synthesis pathway between A- and B-phase syconia. Our research provided the first global analysis of mechanism differences of terpenoid synthesis pathways between A and B phases in *F. hirta* syconia.

## Introduction

In the Angiospermae, more than 90% of flowering plants are pollinated by insects (Kearns et al., 1998). To attract pollinators, many flowering plants release volatile organic compounds (VOCs) from their flowers, inflorescences or specific tissues of inflorescences (Dudareva et al., 2006; Hu et al., 2020). Plants take advantage of VOCs to communicate and interact with their surroundings (Rosenkranz et al., 2021). Mutual adaptation of plant VOCs and insects play a vital role in adaptive evolution. The VOCs perception process of insects is a complex process that the VOCs penetrate pore tubules of the sensillum are bound and dissolved by OBPs and CSPs, transported through the sensillum lymph, and reach the sensory dendrite to activate the membrane-bound OR (Brito et al., 2016).

The *Ficus* species (Moraceae, *Ficus*) and their pollinating fig wasps known today as they form the closest mutually beneficial obligate symbiotic system. Pollinating fig wasps pollinate syconia, and the syconia provide breeding sites for them. For the mutualism, the olfactory attraction of VOCs is key link between the receptive stage (B-phase) syconia and pollinators to maintain this system (Hossaert-McKey et al., 2010). Pre-receptive stage (A-phase) begins with the appearance of the syconium buds. When the syconia are developed to be ready for pollination, B-phase begins. B-phase lasts until fig wasps are attracted by the VOCs, enter the syconia and lay eggs. VOCs attract obligate pollinating fig wasps in the B-phase syconia, while other developmental stages may repel pollinating fig wasps to maintain the specificity between pollinators and host plants (Gu et al., 2012). Therefore, the unique volatile substances were only detected in B-phase syconia may be the main signal to attract pollinators. WpumOBP2 was a major odor binding protein of unique volatile decanal, and *F. pumila* var. *pumila* attract pollinating fig wasps by the binding of decanal with WpumOPB2 in the receptive stage (Wang et al., 2021).

The main components of VOCs in plants are terpenes, fatty acid derivatives, amino acid derivatives and phenylpropane/benzene compounds (Rosenkranz and Schnitzler, 2016). Generally speaking, VOCs is species-specific and different *Ficus* species emit considerably different VOCs composition (Hossaert-McKey et al., 2016). VOCs compounds of five *Ficus* species (*F. benguetensis*, *F. septica*, *F. variegata*, *F. erecta*, and *F. virgata*) were different, which consist mainly of terpenoids and benzenoids (Okamoto and Su, 2021). VOCs components emitted by different development stages were different in the same *Ficus* species. For example, there were significant differences in the compounds of VOCs between A- and B- phase syconia of *Ficus pumila* var. pumila (Wang et al., 2021). Long-term co-evolution makes fig wasps have a preference for specific VOCs emitted by B phase syconia (Hossaert-McKey et al., 2016; Proffit et al., 2020).

The main biosynthetic pathways of VOCs in plants include mevalonic acid (MVA) pathway in the cytoplasm, methylerythritol phosphate (MEP) pathway in the chloroplasts, shikimate pathway and lipoxygenase (LOX) pathway (Dudareva et al., 2013; Rosenkranz and Schnitzler, 2016). Terpenoids are the main compounds of VOCs that are emitted by plants and attract pollinating insects (Soler et al., 2011; Hossaert-McKey et al., 2016). The terpenoid precursors are synthesized by MVA pathway and MEP pathway, including isopentenyl pyrophosphate (IPP), dimethylallyl pyrophosphate (DMAPP), geranyl diphosphate (GPP), geranyl pyrophosphate (GGPP) and farnesyl pyrophosphate (FPP). GPP is a precursor of monoterpenoids (Kumar et al., 2018), FPP is a precursor of sesquiterpenoids (Ma et al., 2019), and GGPP is a precursor of diterpenoids (Liu et al., 2017). Then, terpenoids are synthesized under the catalysis of terpenoid synthases (TPSs) (Dudareva et al., 2013). Structural properties of TPS proteins are drivers of reaction mechanisms leading to the formation of multiple products and underling the molecular evolution of terpene diversity (Tholl, 2006). TPSs form a large family that underlies the diversity of terpenoids (Champagne and Boutry, 2016).

*Ficus hirta* Vahl is a dioecious shrub or small tree that grows in tropical and subtropical regions (Berg, 2003; Yu and Nason, 2013). *F. hirta* bears syconia asynchronously on individual trees (Yu et al., 2006). Roots of *F. hirta* are rich in active ingredients that can be used as medicine and plant-derived popular food (Yi et al., 2013; Wan et al., 2017; Ye et al., 2020). Like other dioecious *Ficus* species, female trees of *F. hirta* bear female syconia that contain female flowers only and produce seeds. Male trees bear male syconia functionally that contain both male and female flowers. The development of syconia of *F. hirta* were also divided into five phases (A-E phases) (Galil and Eisikowitch, 1968; Yu et al., 2006). VOCs emitted by the stomata bracts of syconia were candidate source to attract pollinators over long distances (Hossaert-McKey et al., 1994; Souza et al., 2015; Hu et al., 2020). VOCs emitted by B-phase of *F. hirta* attract obligate pollinators and maintain the obligate mutualism. Terpenoids play important ecological roles in pollinator attraction, allelopathy and plant defense (Mahmoud and Croteau, 2002; Tholl, 2006; Parvin et al., 2014). However, the molecular mechanism differences in terpenoid synthesis pathways between *F. hirta* A-phase and B-phase syconia remains unclear.

Previous studies transcriptomic data was applied to describe VOCs preliminary (Hu et al., 2020). However, transcriptome data only reflect the expression of genes at the transcriptional level. There are many modifications between genes and proteins, which not only affect the expression abundance of proteins directly, but also affect the composition of VOCs. Therefore, these frequent inconsistencies suggest that complementary proteome and VOCs analyses are needed to further be analyzed, including key genes, proteins and metabolites of terpenoid synthesis pathway in syconia bract tissues of different periods. On the basis of comparing the differences of VOCs between A-phase and B-phase syconia, combined transcriptomic and proteomic analysis provide a deeper understanding of the molecular mechanism of how the host *Ficus* species attract obligate pollinators.

In this study, transcriptome sequencing, proteomics and Gas Chromatography-Mass Spectrometer (GC-MS) experiment were used to analyze the difference of genes, proteins and metabolites between A-phase and B-phase syconia, respectively. This study aimed to screen key genes, proteins and metabolites in the terpenoids synthesis pathway to understand the biological process from A-phase to B-phase of *F. hirta* syconia. What is the cause of the differences in terpenoid synthesis pathways between A- and B-phase. The application of multi-omics for jointly analyzing the differences of the terpenoids synthesis pathways in the bracts between A-phase and B-phase. It is helpful to understand the molecular mechanism of maintaining obligate mutualism between *Ficus* species and their pollinator wasps.

## Materials and methods

### Studies species and sample collection

*F. hirta* male syconia were collected at the South China Botanical Garden (SCBG), in Guangdong Province. The area has a subtropical maritime climate, with an annual mean temperature of almost 22°C. 24~36 pre-receptive stage (A-phase) syconia and 24-36 receptive stage (B-phase) syconia were randomly collected from male trees in SCBG, and divided into 3 groups. To avoid pollinator visitation, male syconia were bagged before syconia receptivity. Ostiole bracts were dissected from A-phase and B-phase male syconia without pollinated, and put into Sample Protector for RNA (Takara). RNA sequencing (RNA-seq) was performed on Illumina Hiseq platform.

### Transcriptome data analysis

Transcriptome sequencing data assembly, annotation and differential gene expression analysis had been completed in previous papers (Hu et al., 2020). Both Expectation-Maximization (RSEM) and the most common method Fragments Per Kilobase of transcript sequence per Millions base pairs sequenced (FPKM) were applied to estimate gene expression level (Li and Dewey, 2011). DESeq R package (1.10.1) was used to analyze differential gene expression (*p*-adjusted <0.05 and fold change (FC)>2) (Anders and Huber, 2010). Among the 60,299 unigenes detected in both A- and B-phase of *F. hirta* syconia, 187 (0.31%) differentially expressed genes (DEGs) were found (Hu et al., 2020). Gene Ontology (GO) enrichment of DEGs was analyzed by GOseq R packages. Kyoto Encyclopedia of Genes and Genomes (KEGG) pathways enrichment of DEGs was analyzed using KOBAS (2.0) software (Xie et al., 2011). iTAK software was used to predict plant transcription factors. According to the annotation information of the database, transcription factors and the genes related to terpenoid synthesis pathways were identified, and the relevant information was extracted and sorted out. The heatmap of the target genes expression level was showed by TBtools (Chen et al., 2020).

### Label-free quantitative proteome

Samples of proteome were collected as same as the transcriptome materials. Total protein of tissue samples was extracted by SDT(4%SDS, 10 mM DTT, 100 mM TEAB)-acetone method, and protein precipitate was dissolved with dissolution buffer (8 M Urea, 100 mM TEAB, pH 8.5) (Wu et al., 2014). Draw a standard curve with the absorbance of the standard protein solution and calculate the protein concentration of the sample. Take 20 μg of protein for 12% sodium dodecyl sulfatepolyacrylamide gel electrophoresis (SDS-PAGE). The protein samples were digested by trypsin and then detected by Liquid Chromatography-Mass Spectrometry (LC/MS).

LC/MS analysis was performed using an ultra-nanoflow high-performance liquid chromatography (EASY-nLCTM 1200 nanoscale UHPLC, Thermo Fisher/LC140) system and a Q ExactiveTM series mass spectrometer. The pre-column was a home-made C18 Nano-Trap column (4.5cm×75μm×3μm), and the peptides analytical column was a home-made analytical column (15cm×150μm×1.9μm). Using a Q Exactive™ series mass spectrometer, with Nanospray Flex™ (ESI) ion source, set the ion spray voltage to 2.1 kV and the ion transfer tube temperature to 320°C. The full scan range of the mass spectrometer was m/z 350-1500. The precursor ions with the ion strength of TOP 40 in the full scan were selected for fragmentation by high-energy collisional fragmentation (HCD) method, and secondary mass spectrometry was performed for detection. The peptide fragmentation normalized collision energy was set as 27%. The threshold intensity was 2.2×10^4^, and the dynamic exclusion parameter was 20 s. The raw data of MS detection was named as “.raw”.

The resulting spectrum was searched using Proteome Discoverer 2.2 (PD2.2) according to the Gene Ontology (GO), KEGG and Clusters of Orthologous Groups (COG) (Madzharova and Sabino, 2019). To improve the quality of the analysis results, the PD2.2 software filtered the search results: i) Peptide Spectrum Matches (PSMs) with a reliability of more than 99% were trusted PSMs, and ii) proteins containing at least one unique peptide are trusted proteins, iii) keep only credible spectrum peptides and proteins, and iv) estimate false discovery rate (FDR). Proteomics quality control including peptide length distribution, precursor ion tolerance, unique peptide number, protein coverage, and protein molecular weight. Principal component analysis (PCA) and coefficient of variance (CV) were performed by R packages. The protein quantitation (protein abundance) results were statistically analyzed by *T*-test. The relative quantitative value of each protein in the two comparison samples was tested by *T*-test, and the corresponding *P* value was calculated as the significance index, and the default *P* value was ≤0.05 (Hussain et al., 2021). The up-regulated proteins were screened, when Fold Change ≥ 1.2 and *P* value ≤ 0.05. The down-regulated expression proteins were screened, when Fold Change ≤ 0.83 and *P* value ≤ 0.05. Cell-PLoc 2.0 was used to predict the subcellular localization of proteins in bract tissues (Chou and Shen, 2008). K-mean cluster analysis of DEPs was performed *via* the R package.

GO and interpro (IPR) functional annotation were performed using Interproscan software (Jones et al., 2014). Volcano plot analysis, K-mean cluster analysis, GO, IPR and KEGG were performed for differentially expressed proteins (DEPs) (Huang Da et al., 2009). STRING DB software (http://STRING.embl.de/) was applied for protein interactions analysis (Franceschini et al., 2013).

### VOCs collection and GC-MS analysis

30~35 pre-receptive (A-phase) male syconia and 20-25 receptive (B-phase) male syconia were randomly collected from male trees in SCBG, divided into 3 groups, and put directly into polyethylene terephthalate bags. Before sampling, 120ng/ul mixture of n-nonane and dodecane was added to the sampling tube as the internal standard. Three biological replicates were performed for each stage. The steps of odour collection include, i) putting a syconia into a Teflon film bag with both ends open, ii) fastening one end of the bag with a thin wire, iii) sitting the syconia for 30 minutes, iv) setting the air flow rate at 300 ml/min, v) pumping air into the bag for 5 min and a cycle was completed (Hossaert-McKey et al., 2016). Collect the air in the current sampling environment as a blank control. Samples were stored in a -20°C freezer.

VOCs emitted by syconia were analysed using Gas Chromatography-Mass Spectrometer (GC–MS) system (GCMS-QP2010PLUS). The column used was HP-5MS quartz capillary column (30m × 250μm × 0.25μm). PTV1 injection port need the injection adopts the split mode, and the split ratio set to 10:1. The column temperature was kept at 40°C for 5 minutes in the begin, the increased to 280°C at 7.5°C per minute, and kept for 8 minutes. The inlet temperature was maintained at 40°C for 2.5 minutes. The column flow was set to 2.0 ml/min, the linear velocity was 51 cm/sec, and the purge flow was 3 ml/min. The pre-column pressure was 112 kPa and the injection volume was 2.0 μl. MS conditions were set as ionization by electron bombardment, scanning range 20-45u, electron energy 70 Ev, transmission line temperature 250°C, ion source temperature 230°C. Compounds were mainly identified by matching mass spectra with the standard spectral library (FFNSC1.3, NIST14S, NIST05s) and by comparing Kovats retention indices with that reported in the NIST chemistry Web Book (http://webbook.nist.gov) and published data. The peak area of each compound was quantified as relative quantities of each component based on the normalization method (Soler et al., 2012).

### Data analysis

Each protein has its corresponding transcript, and R packages were used to analyze the correlation between transcriptome and proteome. Correlations were calculated based on Pearson’s statistical method. K-mean cluster heatmap analysis *via* the R package. Key genes, proteins and metabolites involved in terpenoid synthesis pathway in transcriptome, proteomics and VOCs were analyzed and identified according to annotation databases, then using Adobe Illustrator CS6 (AI CS6) to map terpenoid synthesis pathways. The heatmap of the target genes/proteins expression level was showed by TBtools. One-way ANOVA analyzed metabolites data at *P* < 0.05 level and multiple comparisons were performed by *Tukey* test.

## Results

### Transcriptome analysis of bracts in A- and B-Phase of *F. hirta* syconia

**Figure 1:**
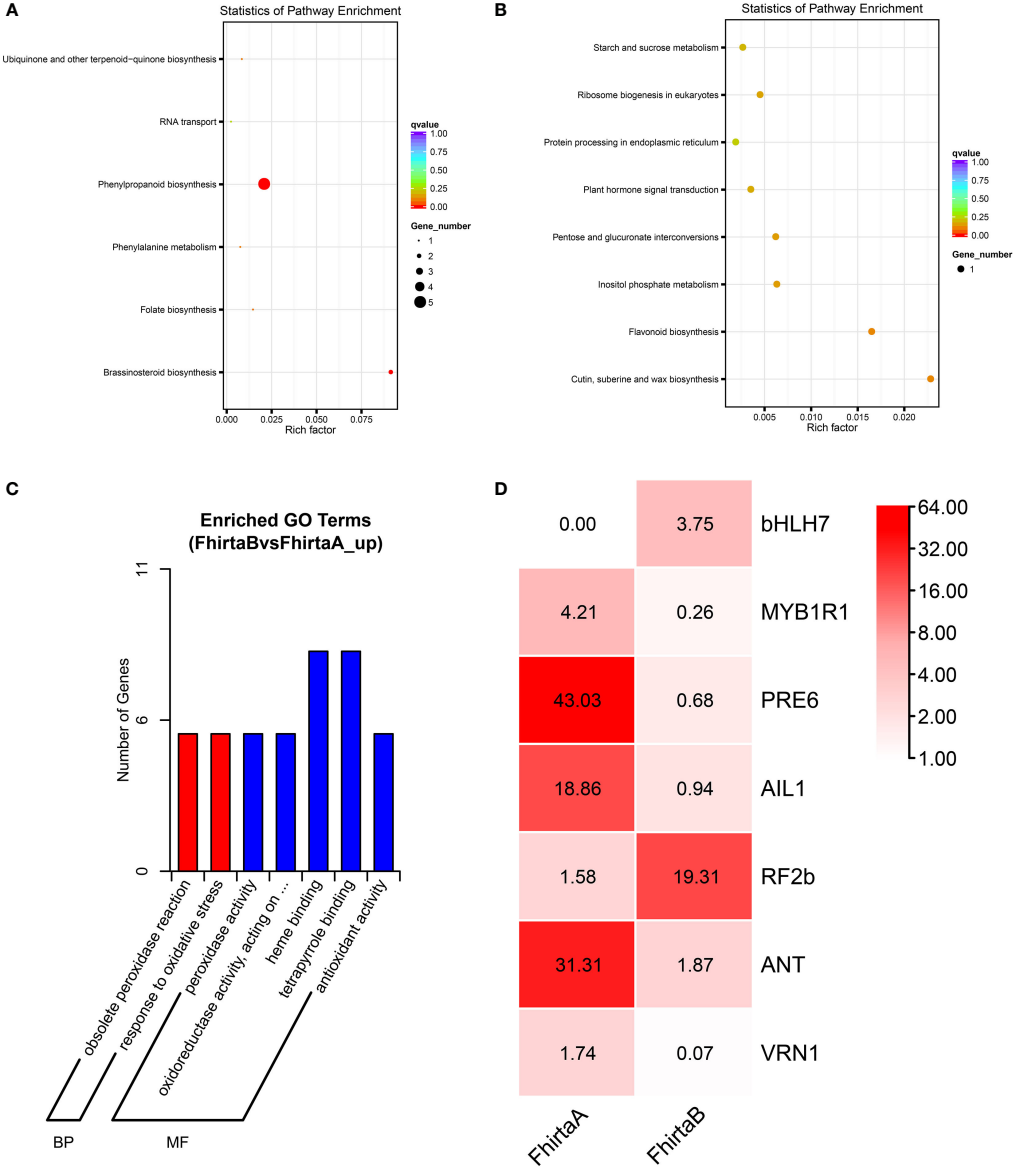
Kyoto Encyclopedia of Genes and Genomes (KEGG) and Gene Ontology (GO) enrichment analysis of differentially expressed genes (DEGs) between A- and B-phase of *F hirta* bracts. **(A)** KEGG classification pathway of up-regulated genes. **(B)** KEGG pathway of down-regulated genes. **(C)** GO classification of up-regulated genes. **(D)** Heatmaps of expression profiles of differentially expressed transcription factors genes according to FPKM. The data inside the box represents FPKM. FhirtaA and FhirtaB represent A phase and B phase of *F hirta* bracts, respectively.

A total of 60,299 unigenes were obtained in both A- and B-Phase syconia, 79 up-regulated genes and 108 down-regulated genes were found to be differently expressed (**Table S1**). The KEGG pathway and GO categorization of 187 differentially expressed genes (DEGs) were analyzed. The KEGG pathways showed the main functions of up-regulated genes were phenylpropanoid biosynthesis, brassinolide biosynthesis, folate biosynthesis, ubiquinone, other terpene quinone biosynthesis, phenylalanine metabolism and RNA transport (**Figure 1A**). The down-regulated genes of KEGG enriched pathways were mainly cutin, suberine and wax biosynthesis, flavonoid biosynthesis, inositol phosphate metabolism, pentose and glucuronate interconversions, ribosome biogenesis in eukaryotes, plant hormone signal transduction, starch and sucrose metabolism, protein processing in endoplasmic reticulum (**Figure 1B**). GO categorization only showed that DEGs were mainly concentrated in molecular functions (e.g. heme binding, tetrapyrrole binding, redox process, peroxidase activity) and biological processes (e.g. peroxidase reaction and oxidative stress response) (**Figure 1C**).

The secondary metabolic process of VOCs were usually regulated by transcription factors, such as *MYB*, *NAC*, *WRKY*, *bHLH*, etc. Therefore, according to the Plant Transcription Factor Database, we analyzed transcription factors from DEGs. The result shown *bHLH7*, *MYB1R1*, *PRE6*(*bHLH163*), *AIL1*, *RF2b*, *ANT*, *VRN1* were significantly differentially expressed. Among them, *bHLH7* and *RF2b* were up-regulated. *MYB1R1*, *PRE6*(*bHLH163*), *AIL1*, *ANT*, *VRN1* were down-regulated (**Figure 1D**). Interestingly, bHLH7 was only expressed in B-phase syconia bract.

### Proteomics analysis of bracts in A- and B-Phase of *F. hirta* syconia

**Figure 2:**
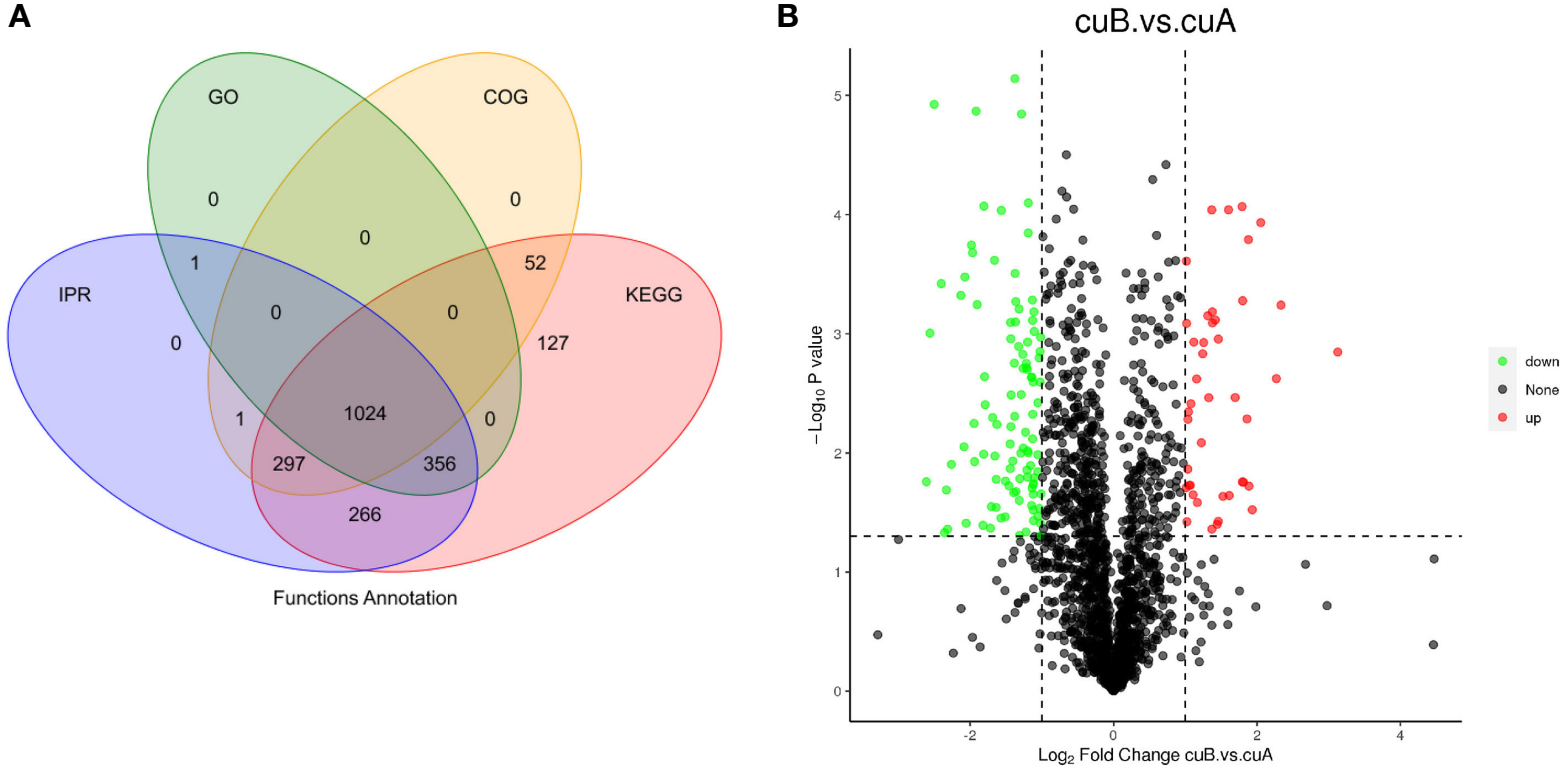
Annotation results and expression profiles of differentially expressed (DEPs) proteins. **(A)** Venn diagram of GO, KEGG, Clusters of Orthologous Groups (COG), Interpro (IPR) database annotation results. **(B)** Volcano maps representing expression pattern of DEGs. Red spots represent upregulated DEGs. Green spots indicate downregulated DEGs. Those shown in black are proteins that did not show obvious changes in A phase and B phase of *F hirta* bracts. CuA VS CuB represent multiples of protein expression in both A phase and B phase of *F hirta* bracts.

The quantitative proteomic was performed to compare the expression differences of proteins between bracts of A- and B-phase syconia. After mass spectrometry data retrieval, the peptide and protein were checked for quality control (**Figure S1**). PC1 represents the score of the experimental group, accounting for 54.48% of the total variation, while PC2 represents repeatability of the experimental group, explaining 18.28% of the total variation (**Figure S2A**). Principal component analysis (PCA) represented a closer association of biological replicates rather than different phases (**Figure S2A**). Coefficient of Variance results showed that CuB samples have better repeatability (**Figure S2B**). A total of 668,534 spectra, with 93,239 matching those of known peptides. Among them, 11,223 specific peptides and 2,729 proteins were identified (**Table S2**). 1,380 proteins were identified in the GO database, 2,122 proteins were identified in the KEGG database, 1,373 proteins were identified in the COG database, and 1,943 domains were identified in the IPR database (**Figure 2A**). A total of 235 significantly DEPs were identified (**Table S3**), of which 57 were up-regulated and 178 were down-regulated (**Figure 2B**).

**Figure 3:**
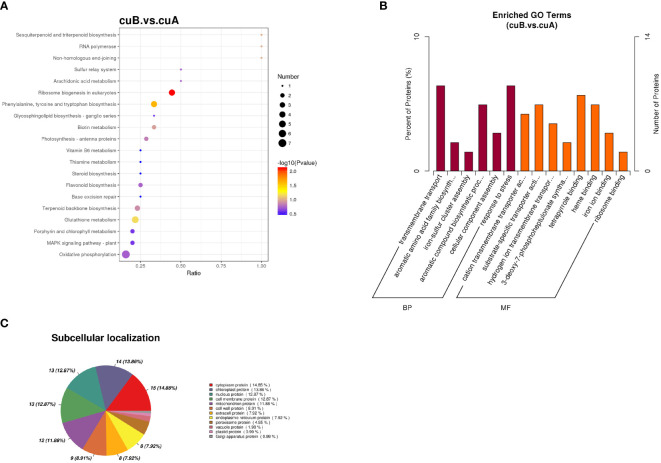
Differential expression proteins (DEPs) enrichment and localization analysis. **(A)** KEGG pathway of DEPs. **(B)** GO enrichment of DEPs. **(C)** Pie chart of subcellular localization of DEPs.

To analyze the functions of DEPs between A- and B-phase, 235 DEPs were mapped into KEGG pathways and GO categorization, respectively. The KEGG pathway enrichments showed that the major enrichment pathways of DEPs were oxidative phosphorylation, glutathione metabolism, terpenoid backbone synthesis, phenylephrine, tyrosine and tryptophan biosynthesis (**Figure 3A**). GO categorization showed that these proteins were mainly concentrated in biological processes such as transmembrane transport and tetrapyrrole binding (**Figure 3B**). The subcellular localization analysis of the DEPs showed that they were mainly located in the cytoplasm and chloroplast (**Figure 3C**).

**Figure 4:**
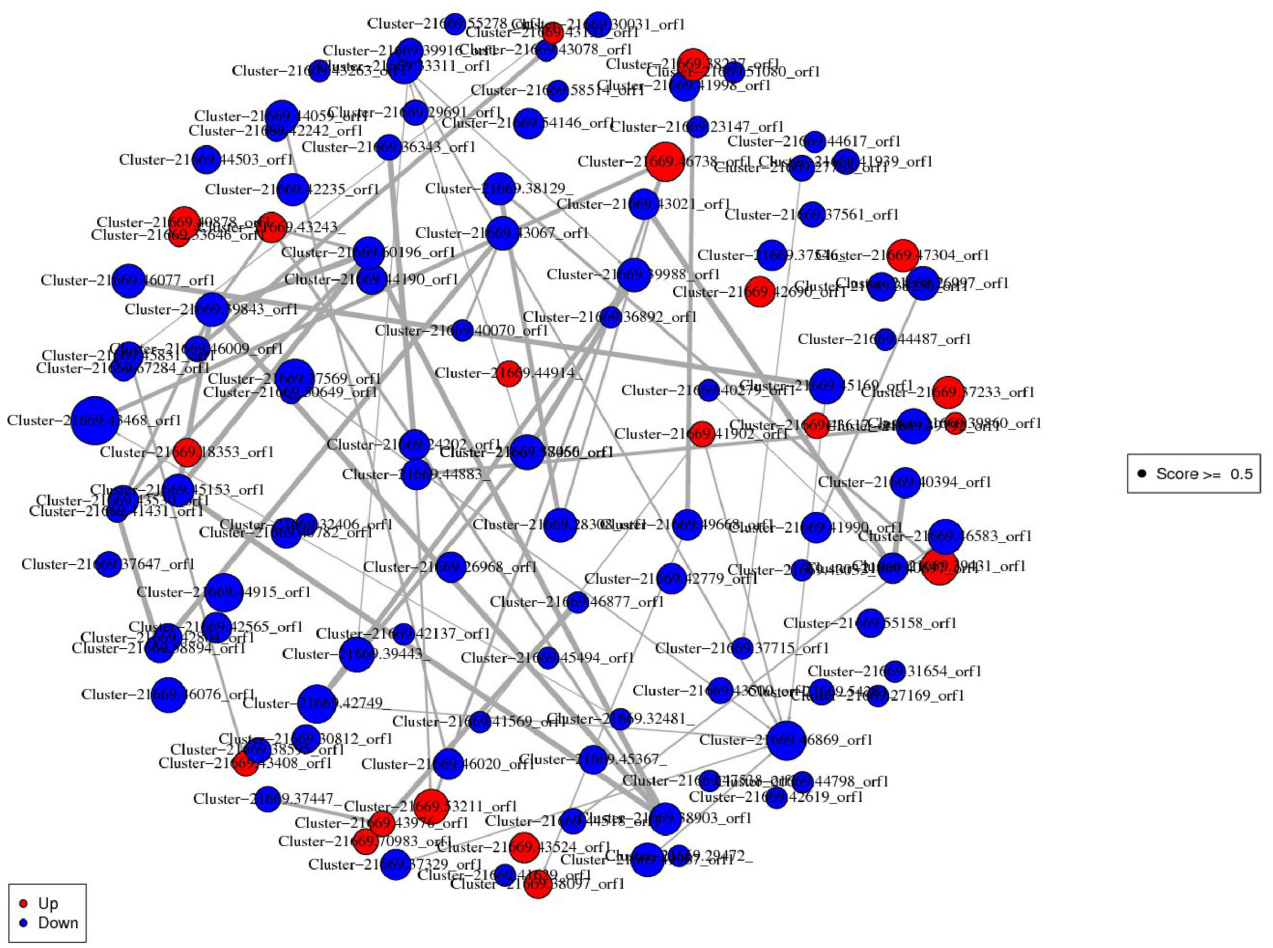
Interaction network diagram of DEPs. Red spots represent upregulated DEGs. Green spots indicate downregulated DEGs.

In the constructed network, cluster-21669.46738_ORF1 (Methionyl-trNA synthetase) was the most important protein up-regulation hub associated with Aminoacyl-tRNA biosynthesis pathway. Cluster-21669.43468_orf1 (Ubiquilin) related to protein processing in endoplasmic reticulum pathway was an important protein down-regulation hub. The DEPs interaction networks and scores were shown in **Figure 4** and **Table S4**. Among them, the interaction analysis of terpenoid synthesis pathway-related proteins showed that Cluster-21669.24202_orf1 (4-diphosphocytidyl methylerythritol kinase, CMK) could interact with Cluster-21669.36343_orf1 (MEP).

### Transcriptome and proteome association analysis

**Figure 5:**
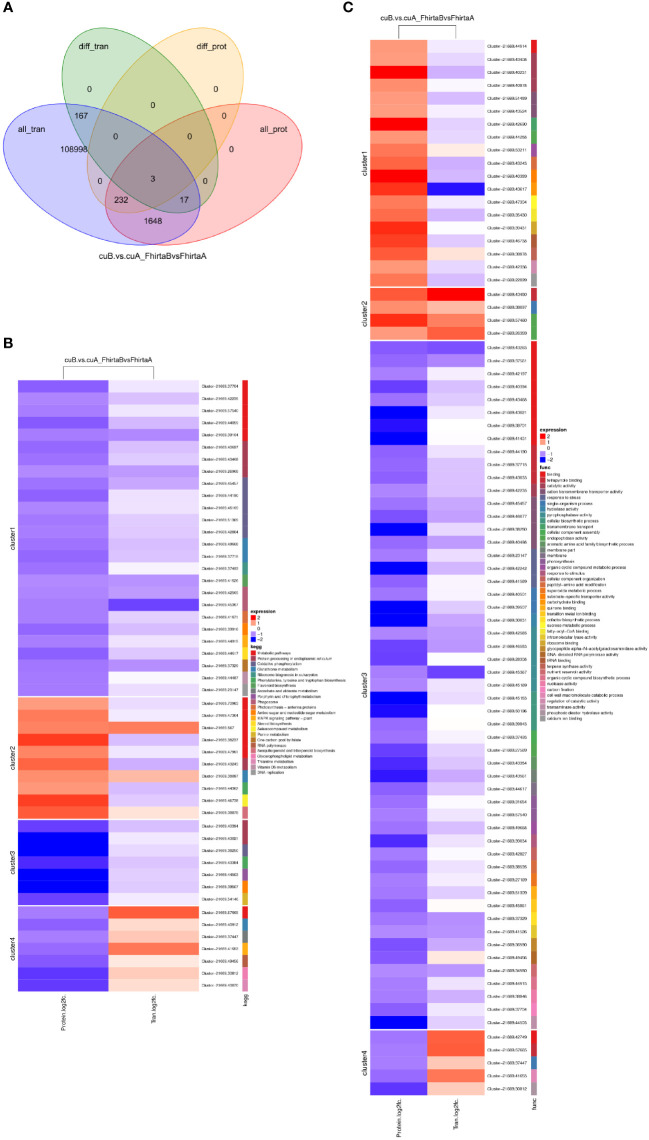
Correlation analysis of transcriptome and proteome. **(A)** Venn diagram of proteins expression and genes expression. **(B)** KEGG pathway of DEGs corresponding to DEPs. Red represents up-regulated and green represents down-regulated. FhirtaA VS FhirtaB represent multiples of transcript expression in both A phase and B phase of *F hirta* bracts. CuA VS CuB represents multiples of protein expression in both A phase and B phase of *F hirta* bracts. Fold Change (FC) represents multiples of genes/proteins expression in two groups. **(C)** GO categorization of DEGs corresponding to DEPs.

It is well known that the relationship between mRNA and protein is complex. Therefore, we performed a correlation analysis of the transcriptome and proteome. Pearson Correlation Coefficient analysis (Pearson Correlation = 0.047) showed that transcriptome was weakly correlated with proteome (**Figure S3**). Transcriptome and proteome were associated with 1082 transcripts/proteins (**Table S5**). Among them, 3 DEGs corresponding to DEPs, namely Cluster-21669.61808 (aspartic protease), Cluster-21669.47934 (mannose-binding lectin), Cluster-21669.43117 (non-specific lipid transfer protein) (**Figure 5A**). Three genes all play an essential role in coping with adversity stress. DEPs of 4 clusters were enriched in 23 KEGG pathways, mainly enriched in the metabolic process. Among them, transcripts/proteins of cluster1 and cluster 3 were both down-regulated (**Figure 5B**). 4 DEPs were enriched in the terpenoid synthesis pathway. DEPs of 4 clusters were enriched in 43 GO processes (**Figure 5C**). These results suggested that there are both complex post-transcriptomic and post-translational modifications in the bracts of syconia from A-phase to B-phase.

### VOCs emitted by male syconia of *F. hirta*

The VOCs of *F. hirta* in A- and B-phase syconia contained more than 60 compounds including 2 fatty acid derivatives, 20 monoterpenes, 44 sesquiterpenes 1 benzenoids, and 1 Nitrogen (**Table S6**). Total of average percent of monoterpenoids emitted by A-phase and B-phase syconia was 8.29% and 37.08%, respectively. Average percent of sesquiterpenes emitted by A-phase and B-phase syconia was 88.43% and 55.02%, respectively. This variation was mainly due to the significant difference in dispersion between A-phase males and B-phase males syconia (*P* = 0.05). Caryophyllene was the most abundant compound emitted by A phase syconia and was the second by B phase syconia. Ocimene was the most abundant compound emitted by B phase syconia and fifth by A-phase syconia. The compounds were detected in B phase syconia but not in A-phase syconia monoterpenes were pinene, camphene, myrcene, camphor, menthol; and the sesquiterpenes were 7-Epi-sesquithujene, farnesene (**Table 1**).

**Table 1:** Volatile compounds monoterpenes and sesquiterpenes emitted by *F. hirta* syconia.

Metabolite name	Normalized amount of volatile compound (%)		
**Monoterpenes**	A Phase	B Phase	
Tricyclene	0.279701 ± 0.093901	0	
Thujene <alpha->	0.106975 ± 0.076027	0.607962 ± 0.190082	
.alpha.-Pinene	0	0.380554 ± 0.620119	
Camphene	0	0.061219 ± 0.031016	
Sabinene	0.420924 ± 0.134895	1.339409 ± 0.804251	
.beta.-Myrcene	0	0.331641 ± 0.142777	
Terpinene alpha	0.111256 ± 0.048199	0.196848 ± 0.086431	
Cymene <para->	0.089512 ± 0.074377	0.159837 ± 0.018086	
D-Limonene	0.142464 ± 0.109211	5.614226 ± 2.811749	
Ocimene <(Z)-, beta->	0.148691 ± 0.031176	0.630069 ± 0.145269	
Ocimene <(E)-, beta->	3.298357 ± 2.106637	20.33461 ± 2.174713	
gamma-Terpinene	2.855996 ± 0.55994	3.084461 ± 0.1595	
unknown 1084	0.035741 ± 0.040984	0.304717 ± 0.152347	
Terpinolene	0.063345 ± 0.014799	0.160307 ± 0.099771	
Linalool	0.191161 ± 0.0889	0.287481 ± 0.021421	
Perillene	0.276044 ± 0.187177	0.062331 ± 0.027489	
1,3,8-p-Menthatriene	0.026553 ± 0.028402	0.500437 ± 0.236772	
Cosmene	0.246179 ± 0.138258	2.6029 ± 0.673882	
Camphor	0	0.246274 ± 0.108866	
Menthol	0	0.172707 ± 0.202493	
**Sesquiterpenes**			
Elemene <delta->	0.495017 ± 0.239381	0.389853 ± 0.02331	
Cubebene <alpha->	1.461843 ± 0.78094	0.370632 ± 0.038567	
Cyclosativene	1.359822 ± 1.427221	0.659959 ± 0.214766	
Copaene <alpha->	11.18039 ± 2.558977	1.925423 ± 0.530602	
Daucene	0.517397 ± 0.096735	0.716682 ± 0.056523	
Bourbonene <beta->	0.114903 ± 0.057929	0.02933 ± 0.003621	
Cubebene <beta->	0.143267 ± 0.011609	0.244524 ± 0.066354	
Elemene <beta->	3.690693 ± 1.060517	0.592203 ± 0.077555	
Sesquithujene <7-epi->	0	0.741083 ± 0.152043	
alpha.-Funebrene	3.460877 ± 2.168216	9.467905 ± 0.882513	
Cedrene <alpha->	1.39091 ± 0.557673	1.520184 ± 0.181372	
Caryophyllene <(E)->	25.1436 ± 12.23496	12.01404 ± 1.182516	
Maaliene <beta->	0.134936 ± 0.040833	0	
unknown 1428	0.952317 ± 0.693508	0.456136 ± 0.063767	
.gamma.-Elemene	0.131153 ± 0.084364	0.128221 ± 0.069312	
Calarene	0.053222 ± 0.026647	0.186355 ± 0.095928	
Isogermacrene D	0.334866 ± 0.288424	0.169613 ± 0.031951	
Guaiene <alpha->	0.673739 ± 0.593171	0.328577 ± 0.082898	
Farnesene <(Z)-, beta->	0	0.292418 ± 0.138151	
Humulene <alpha->	6.136025 ± 0.973794	3.524929 ± 0.271481	
Caryophyllene <9-epi-(E)->	0.659508 ± 0.27048	0.073844 ± 0.127902	
Muurola-4(14),5-diene <cis->	0.633604 ± 0.298112	0.355672 ± 0.100485	
unknown 1469	0.082155 ± 0.030938	0.213291 ± 0.181688	
Muurolene <gamma->	0.948525 ± 0.602066	0.46667 ± 0.198895	
Germacrene D	4.588433 ± 2.555332	3.284334 ± 0.566488	
Acoradiene <beta->	0.069279 ± 0.066496	0.328642 ± 0.091315	
Bicyclogermacrene	0.920439 ± 0.771135	1.678372 ± 1.106348	
Selinene <beta->	0.450857 ± 0.271269	0.235989 ± 0.047297	
Muurolene <alpha->	1.634127 ± 0.821371	2.416825 ± 2.211517	
Amorphene <epsylon->	0.346487 ± 0.019319	0.320938 ± 0.093198	
Cadinene <gamma->	0.917877 ± 0.578696	1.671503 ± 0.317758	
Bulnesene	0.923225 ± 0.507382	0.792212 ± 0.2399	
Bisabolene <(Z)-, alpha->	0.068381 ± 0.09625	0.080953 ± 0.055912	
Cadinene <delta->	1.852318 ± 0.221276	1.057476 ± 0.186988	
Cadinene <alpha->	0.171423 ± 0.038027	0.137721 ± 0.023957	
Bisabolene <(E)-, gamma->	0.178962 ± 0.119737	0.321178 ± 0.049371	
Calacorene <alpha->	0.2378 ± 0.050368	0.173671 ± 0.031566	
Germacrene B	0.173741 ± 0.105013	0.188113 ± 0.069762	
Calacorene <beta->	0.047576 ± 0.008562	0.063438 ± 0.025872	
Cedrene <alpha-, epoxy->	8.765918 ± 12.64395	3.92387 ± 0.84527	
Cedranone	3.272034 ± 2.72078	1.367145 ± 0.280374	
Isolongifolen-5-one	0.577825 ± 0.418587	0.117931 ± 0.059512	
.tau.-Cadinol	2.86869 ± 2.108536	1.706591 ± 0.526422	
9-Cedranone	0.661865 ± 0.63051	0.287667 ± 0.096888	

Normalized amount of volatile compound = (peak area of volatile compound)/(total peak area of all volatile compounds). Values represent means of three independent determinations.

### Key genes, proteins and metabolites involved in terpenoid synthesis pathway in *F. hirta*

**Figure 6:**
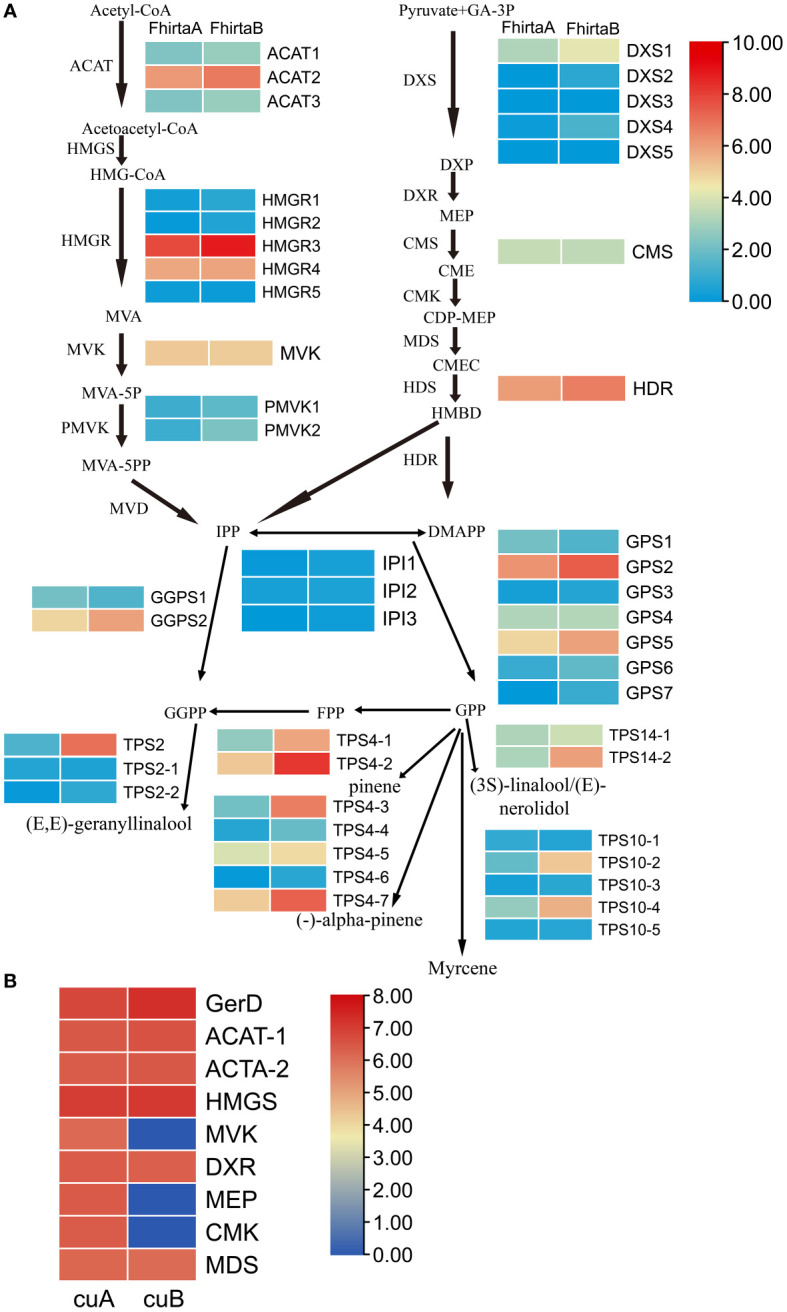
Expression profiles of genes and proteins involved in the terpenoid synthesis pathway of *F hirta*. **(A)** Expression levels for genes in A- and B-phase of *F hirta* bracts were shown by heatmap according to log_2_ (FPKM). **(B)** Expression levels for proteins in A- and B-phase of *F hirta* bracts were shown by heatmap according to log_10_ (Protein Abundance). CuA and CuB represent A phase and B phase of *F hirta* bracts, respectively.

29 candidate enzyme genes were involved in the pathway of terpenoid synthesis precursors and 20 terpenoid synthases (TPSs) were identified in the transcriptome (**Table S7**). For the terpenoid synthesis pathway, except for 4-diphosphocytidyl methylerythritol synthase (CMS) and mevalonate kinase (MVK), at least one transcript of candidate enzyme genes was up-regulated in B-phase compared with *F. hirta* A-phase. Acetyl-CoA C-acetyltransferase protein (*ACAT2*), 3-hydroxy-3-methylglutaryl-coenzyme A reductase (*HMGR3*), geranylgeranyl diphosphate synthase (*GGPS2*), 4-hydroxy-3-methylbut-2-enyl diphosphate reductase (*HDR*), geranyl diphosphate synthase (*GPS2*), *TPS2*, *TPS4-2*, *TPS4-3*, *TPS4-7*, *TPS14*, and *TPS10-4* were highly expressed in *F. hirta* B-phase Syconia (**Figure 6A**).

A total of 9 terpenoid synthesis proteins were identified in the proteome. Among them ACAT and hydroxymethylglutaryl-CoA synthase (HMGS) proteins in the MVA pathway were up-regulated, while 1-deoxy-D-xylulose 5-phosphate reductoisomerase (DXR), 4-(cytidine 5’-diphospho)-2-c-methyl-d-erythritol kinase (CMK), 2-C-methyl-D-erythritol 2,4-cyclodiphosphate synthase (MDS) and MEP in the MEP pathway were down-regulated (**Figure 6B**).

20 monoterpenes and 44 sesquiterpenes were detected from *F. hirta* A-phase and B-phase Syconia (**Table S6**). We found pinene, myrcene and linalool contents were higher in the VOCs of B-phase syconia than in those of A-phase (**Figure 6A** and **Table S6**). The change of these metabolites content was consistent with the expression pattern of the corresponding *TPSs* (*TPS4*, *TPS10*, *TPS14*) gene.

## Discussion

In previous research, it was speculated that phenylpropanoid and terpenoids were the main enriched VOCs components of *F. hirta* B-phase syconia by analyzing the genes related to VOCs (Hu et al., 2020). Terpenoids play ecological roles in pollinator attraction, allelopathy, and plant defense (Mahmoud and Croteau, 2002; Tholl, 2006). For the first time, we used a comprehensive analysis of transcriptome, proteome, and metabolism to understand the differences of the terpenoids synthesis pathways in the bracts of both A-phase and B-phase in *F. hirta*. It is important for us to understand molecular mechanism of maintaining obligate mutualism between the *Ficus* species and their pollinator wasps.

The VOCs emitted by syconia were mainly composed of terpenoids, which play ecological roles in pollinator attraction, allelopathy, and plant defense (Mahmoud and Croteau, 2002; Tholl, 2006; Parvin et al., 2014). Terpenoids were predominant in VOCs emitted by both A-phase and B-phase in *F. hirta* (**Table S6**). Most of the monoterpenoids were emitted by B-phase syconia higher than the A-phase, while most of sesquiterpenes were lower (**Table 1**). Monoterpenes Ocimene, D-Limonene, gamma-Terpinene were important component of VOCs emitted by B phase syconia (**Table 1**). β-myrcene, E-β-ocimene and D-limonene were elicited by *Mimulus lewisii* flowers evoke significant neural responses in bumblebee. Besides, a synthetic blend of the three chemical compound evoke the same responses as natural scents (Byers et al., 2014). The monoterpenes that were only detected in the VOCs of B-phase syconia were pinene, camphene, myrcene, camphor, menthol (**Table 1**). Compared with A-phase, the unique volatile monoterpenes emitted by B-phase syconia may play an important role in attracting pollinating fig wasps. Monoterpenoids, such as linalool, camphene, and cineole, were involved in pollinator attraction and allelopathy (Mahmoud and Croteau, 2002). The olfactory response experiment of weevil showed that camphene had an attracting effect on them (Sharaby and Al-Dosary, 2014). The olfactory receptor neuron of the pine weevil exhibited a strong response to α-pinene (Wibe et al., 1998). The olfactory responses of *Mahanarva spectabilis* to forage VOCs suggested that menthone, eucalyptol and camphor were all compounds likely to cause loss of attractiveness or repellence (Silva et al., 2019). Menthol exhibited moderate repellent effects on *Drosophila suzukii* (Corda et al., 2020). Compared with A-phase, the unique decanal in *Ficus pumila* var. *pumila* B-phase attracts pollinating fig wasps (Wang et al., 2021). We speculated that pinene, camphene, myrcene, camphor, and menthol play important roles to attract obligate pollinating fig wasps. Caryophyllene, funebrene, cedrene were main component of volatile sesquiterpenes emitted by B phase syconia (**Table 1**). (E)-caryophyllene was significantly increased in the damaged plants (Riffel et al., 2021). Red-rot disease infected plants release greater amounts of (+)-β- funerene than herbivore-infected plants (Peñaflor and Bento, 2018). Cedrene accumulated after whitefly infects tobacco, conferring resistance to the whitefly (Luan et al., 2013). We suspect that the emitting of caryophyllene, funebrene and cedrene by B phase syconia are related to plant defense. In conclusion, monoterpenoids mainly attract pollinators, while sesquiterpenoids were related to plant defense response in *F. hirta*. If we want to know what attracts pollinating wasps, we need to perform experiments of electroantennographic detection coupled with gas chromatography (GC-EAD) and Y-tube olfactometer tests for identification. Combine these results and our results suggested that B-phase syconia need to produce more monoterpenoids and reduce the accumulation of sesquiterpenes to attract pollinators in *F. hirta*.

For terpenoids synthesis, transcription factors activate/repress their activities by binding to homeopathic elements in the promoter region of target gene, then regulate metabolic pathways. At the transcriptional level, the expression levels of *ACAT2*, *HMGR3*, *GGPS2*, *HDR*, *GPS2*, *TPS2*, *TPS4-2*, *TPS4-3*, *TPS4-7*, *TPS14*, *TPS10-4* increased in the bracts in B-phase (**Figure 6A**). Interestingly, pinene, myrcene and linalool were also higher in B-phase syconia than in those by A-phase (**Table S6**). This result indicates that up-regulation of *TPSs* gene expression can directly increase the content of related metabolites. Seven differentially expressed genes were screened from the transcriptome, *bHLH7*, *MYB1R1*, *PRE6* (*bHLH163*), *AIL1*, *RF2b*, *ANT*, and *VRN1* (**Figure 1D**). Transient expression of *AabHLH1* in *Artemisia annua* leaves increased the transcript level of *HMGR* (Ji et al., 2014). Transcription factors *MYB*, *NAC*, *ARF*, *WRKY*, *MYC*, *ERF* and *GRAS* were co-expressed with terpenoid biosynthesis genes, which may regulate terpenoid biosynthesis (Yu et al., 2021). *HY5*, a member of the *Arabidopsis* bZIP family of transcription factors, mediated the regulation of the terpene synthase AtTPS03 (Michael et al., 2020). MYB21 and MYC2 complex regulated *FhTPS1* expression in *Freesia hybrida* and *Arabidopsis* (Yang et al., 2020). Transcription factor *CitERF71* activated the terpene synthase gene *CitTPS16* by binding to the ACCCGCC and GGCGGG motifs of promoter, and was involved in the synthesis of E-geraniol in sweet orange fruit (Li et al., 2017). EREB58 can bind to the GCC-box in the promoter region of *TPS10* to promote its expression (Li et al., 2015). According to research progress in other plants and Plant Transcription Factor Database (http://planttfdb.gao-lab.org/index.php), we speculated *RF2b*, *VRN1* (Loukoianov et al., 2005), *ANT*, *PRE6* (Ferrero et al., 2019) and *MYB1R1* (Wang et al., 2016) were involved in regulating growth and development, and *AIL1* were involved in regulating ethylene signaling pathway. *bHLH7* may regulate metabolic processes (Chen, 2018). The differences in terpenoid synthesis pathways between A- and B-phase may be mainly caused by transcription factors regulating the expression of key enzyme genes, thereby regulating the synthesis of terpenoids. Interestingly, bHLH7 was not expressed in A-phase syconia bract but in the B phase. Based on this, we speculate that a key transcription factor *bHLH7* may regulate the expression of key enzymes involved in the terpenoid synthesis pathway in B-phase syconia.

In proteome, ACAT and HMGS in the MVA pathway were up-regulated, while DXR, CMK, MDS and MEP in the MEP pathway were down-regulated (**Figure 6B**). When one pathway is blocked with specific inhibitors, compensation can be observed with precursors produced by the other pathway (Henry et al., 2015; Mendoza-Poudereux et al., 2015). These results suggested that, at the protein level, the synthesis of terpenoids precursors in the B-phase of syconia were mainly synthesized through the MVA pathway.

In addition, there were three differentially expressed transcript/proteins that were related to adversity stress, namely Cluster-21669.61808 (aspartic protease), Cluster-21669.47934 (mannose-binding lectin), and Cluster-21669.43117 (non-specific lipid transfer protein) in transcriptome and proteome (**Figure 5A**, **Tables S3**, **S5**). The different developmental phases of syconia bracts involve many physiological and biochemical processes regulated by genes (Zhang et al., 2020; Wang et al., 2021). Aspartic proteinase was involved in protein processing and degradation, male and female gamete development, and played a vital role in plant coping with adversity stress (Huang et al., 2013; Scandola and Samuel, 2019; Soares et al., 2019). Mannose-binding lectin was important in controlling pests and diseases, resisting pathogenic microorganisms, and resisting higher herbivorous animals (Chen et al., 2021). The pepper mannose-binding lectin gene *CaMBL1* played a vital role in regulating cell death and defense responses to microbial pathogens (*Pseudomonas syringae* pv tomato, *Alternaria brassicicola*) (Hwang and Hwang, 2011). Overexpression of *OsJRL* (jacalin-related mannose-binding lectin) enhanced the salt tolerance and increased the expression levels of many stress-related genes in rice (He et al., 2017). Non-specific lipid-transfer proteins were involved in biotic stress, abiotic stress, and various metabolic processes (Tomassen et al., 2007; Liu et al., 2015; Gangadhar et al., 2016). These results indicate that many metabolic processes occurred in the bracts of A- and B-phase. And these processes produce metabolites in response to adversity stress, which provide a suitable environment for *F. hirta* B-phase syconia to attract pollinator fig wasps.

These findings contribute to understand the mechanism differences of terpenoids synthetic pathways in A- and B-phase syconia. Langenheim (1994) argued that terpenoids may be factors determining some properties of terrestrial plant communities and ecosystems. Higher plant terpenoids were closely related to many ecologically relevant characteristics (plant pollination, direct plant defense, allelopathy, formation of reactive gases in troposphere) (Langenheim, 1994; Mahmoud and Croteau, 2002; Tholl, 2006; Rosenkranz et al., 2021). Studying the mechanism differences of terpenoids synthetic pathways in both A- and B-phase syconia is important for further understanding the obligate mutualism and ecological implications.

## Data availability statement

The transcriptome data presented in the study are deposited in the NCBI's Short Read Archive (SRA) repository, accession number PRJNA491590; The proteome data presented in the study are deposited in the integrated proteome resources repository, accession number IPX0003971000 (https://www.iprox.cn/page/project.html?id=IPX0003971000).

## Author contributions

SF, YJ and WL performed the experiments. SF and RW analyzed the data. FS, XC and HY wrote the paper. All authors read and approved the final paper.

## Funding

This work was supported by the National Natural Science Foundation of China (Grants Nos. 31971568).

## Acknowledgments

We thank Liao Yao-lin and Cheng Yu-fen for guidance on the GC-MS experiment. We also thank Novogene for guidance on proteome of *Ficus hirta* Vahl bracts.

## Conflict of interest

The authors declare that the research was conducted in the absence of any commercial or financial relationships that could be construed as a potential conflict of interest.

## Publisher’s note

All claims expressed in this article are solely those of the authors and do not necessarily represent those of their affiliated organizations, or those of the publisher, the editors and the reviewers. Any product that may be evaluated in this article, or claim that may be made by its manufacturer, is not guaranteed or endorsed by the publisher.

## Supplementary material

Supplementary Figure 1 Protein qualitative data quality control. **(A)** Diagram of protein coverage distribution. **(B)** Diagram of precursor ions tolerance distribution. **(C)** Statistical diagram of protein molecular weight. **(D)** Diagram of unique peptide distribution.

Supplementary Figure 2 All samples were analyzed by principal component analysis (PCA) and coefficient of variance (CV). **(A)** PCA of the proteome data in a 2D graph of PC1 and PC2. The plot shows the effect for A-phase (CuA), B-phase (CuB). CuA and CuB represent A phase and B phase of *F. hirta* bracts, respectively. **(B)** Display of reproducibility CV of all samples.

Supplementary Figure 3 Pearson Correlation Coefficient analysis of the transcriptome and proteome. FhirtaA VS FhirtaB represent multiples of transcript expression in both A phase and B phase of F. hirta bracts. CuA VS CuB represent multiples of protein expression in both A phase and B phase of *F. hirta* bracts.
