# Supplementary figures and images for: Multi-omics analysis the differences of VOCs terpenoid synthesis pathway in maintaining obligate mutualism between Ficus hirta Vahl and its pollinators
Source: Front Plant Sci. 2022 Nov 15;13:1006291. doi: 10.3389/fpls.2022.1006291 (PMC9707799; doi:10.3389/fpls.2022.1006291)

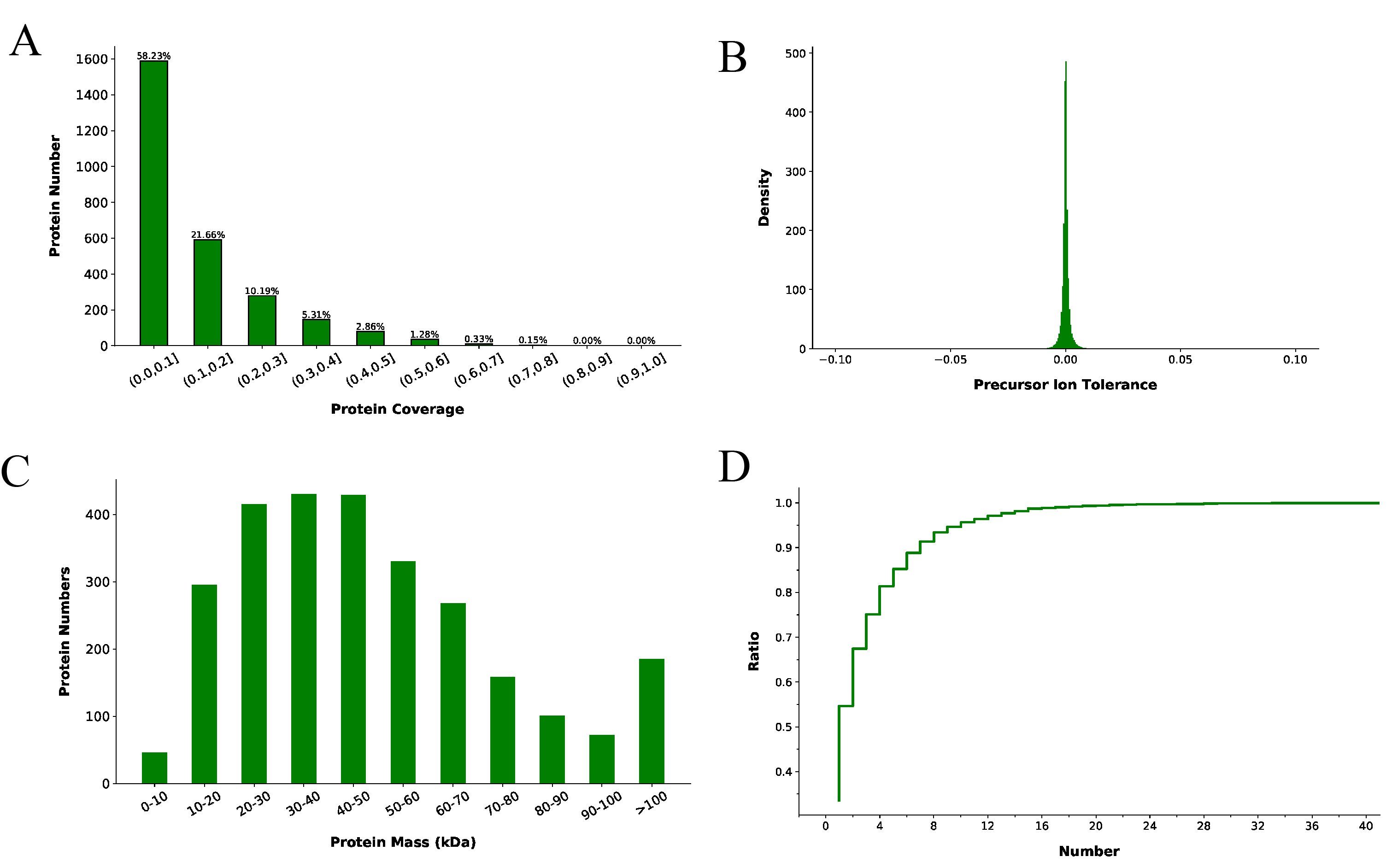

Supplement: Supplementary Figure 1 — Protein qualitative data quality control. (A) Diagram of protein coverage distribution. (B) Diagram of precursor ions tolerance distribution. (C) Statistical diagram of protein molecular weight. (D) Diagram of unique peptide distribution. [file Image_1.jpeg]

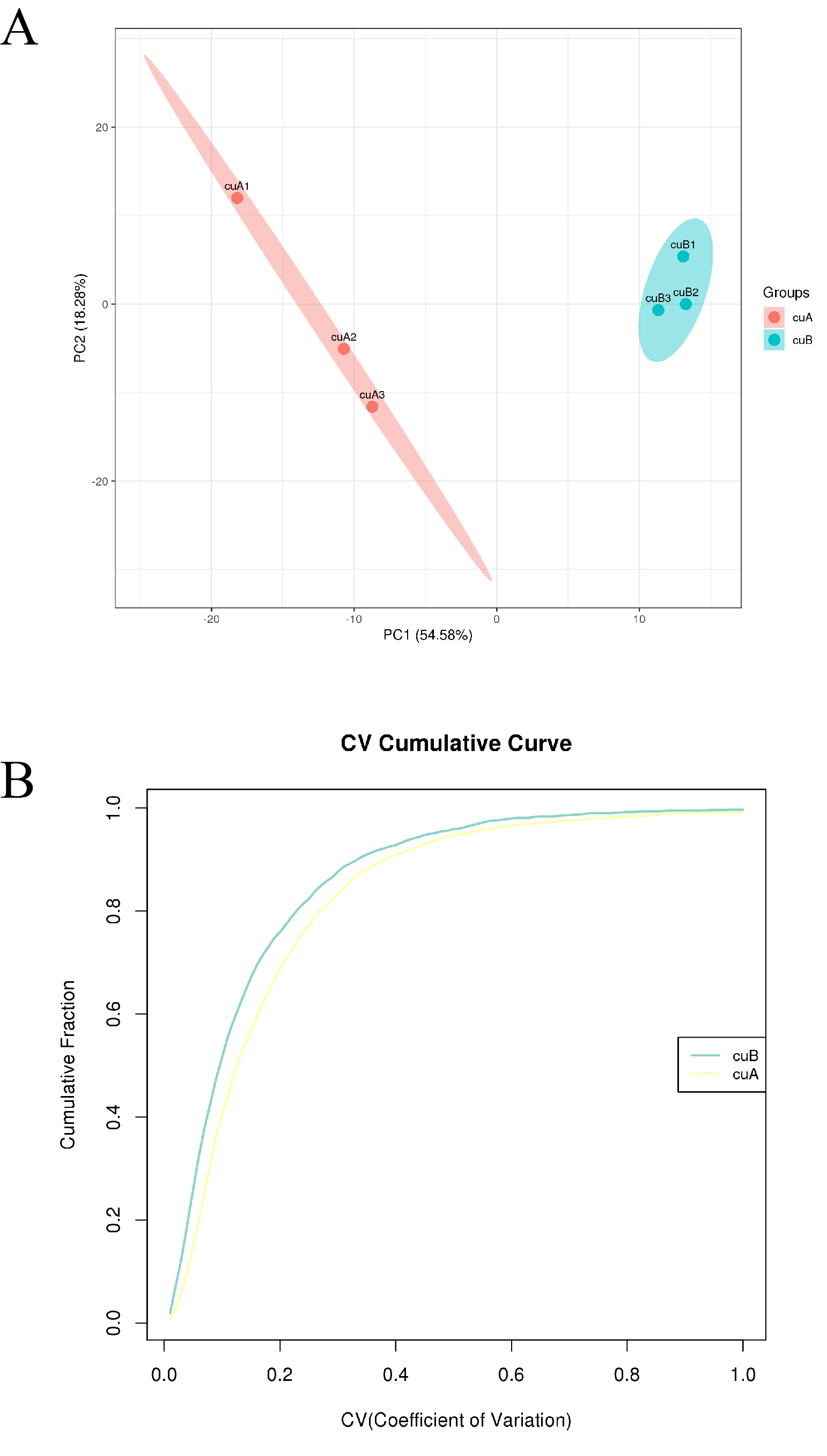

Supplement: Supplementary Figure 2 — All samples were analyzed by principal component analysis (PCA) and coefficient of variance (CV). (A) PCA of the proteome data in a 2D graph of PC1 and PC2. The plot shows the effect for A-phase (CuA), B-phase (CuB). CuA and CuB represent A phase and B phase of F. hirta bracts, respectively. (B) Display of reproducibility CV of all samples. [file Image_2.jpeg]
